# Relationship between the transcriptional expression of PIM1 and local control in patients with head and neck squamous cell carcinomas treated with radiotherapy

**DOI:** 10.1007/s00405-021-07223-4

**Published:** 2022-01-06

**Authors:** Xavier León, Jacinto García, Albert Pujol, Julia de Juan, Rosselin Vásquez, Miquel Quer, Mercedes Camacho

**Affiliations:** 1 Otorhinolaryngology Department, Hospital de La Santa Creu I Sant Pau, Universitat Autònoma de Barcelona, C/Mas Casanovas, 90, 08041 Barcelona, Spain; 2 UVIC. Universitat Central de Catalunya, Barcelona, Vic Spain; 3 Centro de Investigación Biomédica en Red de Bioingeniería, Biomateriales Y Nanomedicina (CIBER-BBN), Madrid, Spain; 4 Otorhinolaryngology Department, Hospital del Mar, Universitat Autònoma de Barcelona, Barcelona, Spain; 5 Genomics of Complex Diseases, Research Institute Hospital Sant Pau, IIB Sant Pau, Barcelona, Spain

**Keywords:** PIM-1, Head and neck carcinoma, Radiotherapy, Local control, Biomarker

## Abstract

**Purpose:**

Proviral integration site for Moloney murine leukemia virus (PIMs) are proto-oncogenes encoding serine/threonine kinases that phosphorylate a variety of substrates involved in the regulation of cellular processes. Elevated expression of PIM-1 has been associated with poor prognosis in several types of cancer. There are no studies that have analyzed the response to radiotherapy in patients with head and neck squamous cell carcinoma (HNSCC) according to the expression of PIM-1. The aim of our study was to analyze the relationship between the transcriptional expression of PIM-1 and local response to radiotherapy in HNSCC patients.

**Methods:**

We determined the transcriptional expression of PIM-1 in 135 HNSCC patients treated with radiotherapy, including patients treated with chemoradiotherapy (*n* = 65) and bioradiotherapy (*n* = 15).

**Results:**

During the follow-up, 48 patients (35.6%) had a local recurrence of the tumor. Patients with local recurrence had a higher level of PIM-1 expression than those who achieved local control of the disease (*P* = 0.017). Five-year local recurrence-free survival for patients with a high expression of PIM-1 (*n* = 43) was 44.6% (95% CI 29.2–60.0%), and for patients with low expression (*n* = 92) it was 71.9% (95% CI 62.5–81.3%) (*P* = 0.007). According to the results of multivariate analysis, patients with a high PIM-1 expression had a 2.2-fold increased risk of local recurrence (95% CI 1.22–4.10, *P* = 0.009).

**Conclusion:**

Patients with elevated transcriptional expression levels of PIM-1 had a significantly higher risk of local recurrence after radiotherapy.

**Supplementary Information:**

The online version contains supplementary material available at 10.1007/s00405-021-07223-4.

## Introduction

Radiotherapy is one of the therapeutic options for patients with head and neck squamous cell carcinoma (HNSCC). After treatment with radiotherapy, a variable percentage of patients, depending on the location and extension of the tumor, have a local recurrence. The availability of biomarkers with the capacity to discriminate tumor radiosensitivity would make it possible to offer radiotherapy to those patients with tumors with a greater probability of response, reducing the percentage of local recurrence and the need for salvage surgery.

Proviral integration site for Moloney murine leukemia virus (PIMs) are proto-oncogenes encoding serine/threonine kinases that phosphorylate a variety of substrates involved in the regulation of cellular processes such as proliferation, survival, apoptosis, migration, and cell cycle regulation. The PIM family consists of three members, PIM-1, PIM-2, and PIM-3 [1].

Elevated expression of PIM-1 has been associated with poor prognosis in patients with leukemia [2] or lymphoma [3], as well as in patients with squamous cell carcinomas located in the esophagus [4] or the lung [5], or adenocarcinomas located in the breast [6], lung [5], pancreas [7], colon [8], stomach [9] or prostate [10].

Studies in experimental models of lung carcinoma [11] or pancreatic carcinoma [12] have shown that PIM-1 expression is associated with a reduction in radiosensitivity. In a study performed with HNSCC cell lines, Peltola et al. [13] demonstrated that PIM-1 expression protects tumor cells from radiotherapy-induced damage. To our knowledge, there are no studies that have analyzed the response to radiotherapy in patients with HNSCC according to the expression of PIM-1.

The aim of our study was to evaluate the relationship between PIM-1 transcriptional expression and sensitivity to radiotherapy in a cohort of patients with HNSCC.

## Materials and methods

### Patients

We carried out a retrospective study based on the analysis of biopsies obtained from the primary location of the tumor prior to any type of treatment in a cohort of 135 patients with a histologically confirmed squamous cell carcinoma located in the oropharynx, hypopharynx or larynx, and treated with radiotherapy during the period 2008–2016. Patients who received concomitant treatment with chemotherapy (chemoradiotherapy) or cetuximab (bioradiotherapy) were also included in the study. Clinical data were obtained from a database that prospectively collects information on all patients with a malignant head and neck tumor treated at our center since 1985 [14].

All patients included in the study were evaluated by the Oncologic Committee of our institution, which proposed the treatment according to the center's clinical guidelines. Table 1 shows the characteristics of the patients included in the study. Given the interaction in tobacco and alcohol consumption, a combined variable of toxics consumption was created with three categories: no consumption; moderate consumption (< 20 cigarettes/day and/or  < 80 g alcohol/day); and severe consumption (≥ 20 cigarettes/day and/or  ≥ 80 g alcohol/day). The HPV status of patients with oropharyngeal carcinoma was determined by viral DNA detection with SPF-10 RT-PCR, using the LiPA25_vl reverse hybridization assay for genotyping. The loco-regional extension category of patients with HPV-positive oropharyngeal tumors was reclassified according to the 8th edition of the TNM.

**Table 1:** Characteristics of the patients included in the study

	*N* (%)
Mean age (standard deviation) years	63.6 (11.7)
Gender	
Men	120 (88.9)
Women	15 (11.1)
Age	
<65 years	75 (55.6)
≥65 years	60 (44.4)
Toxic consumption	
None	14 (10.4)
Moderate	20 (14.8)
Severe	101 (74.8)
Location	
Oropharynx	63 (46.7)
Hypopharynx	16 (11.8)
Larynx	56 (41.5)
Local extension	
cT1–T2	74 (54.8)
cT3–T4	61 (45.2)
Regional extension	
cN0	81 (60.0)
cN1	22 (16.3)
cN2	30 (22.2)
cN3	2 (1.5)
Histologic grade	
Well differentiated	13 (9.6)
Moderately differentiated	109 (80.7)
Poorly differentiated	13 (9.6)
Treatment	
Radiotherapy	55 (40.7)
Chemoradiotherapy	65 (48.2)
Bioradiotherapy	15 (11.1)

Treatment with radiotherapy consisted of the administration of a dose of 70–72 Gy on the primary location of the tumor, 50 Gy on the lymph node areas at risk in cN0 patients, and 70–72 Gy on the affected lymph node areas in cN + patients. Radiotherapy planning was done using 3D conformal fields until 2010, and IMRT (Instensity_Modulated Radiation Therapy) from 2011 onwards, with a linear accelerator as the radiation source. Most patients were treated with standard fractionation (2 Gy/fraction, 1 fraction/day, 5 days/week). Thirteen patients (9.6%) followed a hyperfractionation regimen (1.2 Gy/fraction, 2 fractions/day, 5 days/week). Sixty-five patients had a concomitant treatment with chemoradiotherapy and 15 bioradiotherapy with cetuximab. Patients treated with chemoradiotherapy received between two and three cycles of cisplatin at a dose of 100 mg/m^2^ every three weeks (*n* = 55), or carboplatin 1.5 AUC every week (*n* = 10). Those treated with bioradiotherapy received cetuximab 400 mg/m^2^ on day 1 of the week preceding radiotherapy, and a weekly dose of 250 mg/m^2^ cetuximab during radiotherapy. Seventeen patients were treated with uni or bilateral neck dissections after completing radiotherapy.

All patients included in the study had a follow-up of more than 2 years. The mean follow-up period was 5.4 years (standard deviation 3.3 years).

The study was approved by the Institutional Review Committee and was conducted following the principles established in the Declaration of Helsinki.

### Transcriptional analysis

The biopsy samples obtained from each patient were immediately included in RNA-later (Quiagen GmbH, Hilden, Germany) to prevent RNA degradation and stored at −80 °C until processing. Total RNA was extracted using Trizol (Invitrogen, Carlsbad, USA) according to the manufacturer's instructions. The cDNA was obtained by reverse transcription of 1 µg of RNA with the High-Capacity cDNA Archive Kit (Applied Biosystems, Foster City, USA), and the transcriptional expression of PIM-1 and beta-actin as an endogenous control was evaluated by RT-PCR in an ABI Prism 7000 using validated pre-designed assays (TaqMan Gene Expression Assays; Applied Biosystems).

### Statistical analysis

We compared the expression levels of PIM-1 according to gender, age, toxics consumption, location of the primary tumor, local and regional extension of the tumor, histological grade, and the local control of the tumor after treatment with radiotherapy. The distribution of the PIM-1 expression values did not meet the normality criteria, hence the non-parametric techniques of Mann–Whitney and Kruskal–Wallis were used in the comparison of the PIM-1 expression levels. For patients with oropharyngeal tumors, PIM-1 expression was analyzed in relation to the HPV status. The continuous value of PIM-1 expression was categorized according to the local disease control after treatment with radiotherapy with a recursive partitioning analysis, using the CRT (Classification and Regression-Tree) model. We calculated the local recurrence-free survival according to the categories defined by the recursive partitioning analysis with the Kaplan–Meier method, using the log-rank test in the comparison of the survival curves. A multivariate analysis was carried out with the Cox proportional hazards model, considering the local recurrence-free survival as the dependent variable, and the location of the primary tumor, the local (cT1-2 versus cT3-4) and regional (cN0 versus cN +) extension of the tumor, the histological grade, as well as the expression category of PIM-1 as independent variables.

## Results

There were no significant differences in the transcriptional expression of PIM-1 according to sex (*P* = 0.941), age *(P* = 0.060), toxics consumption (*P* = 0.301), location of the primary tumor (*P* = 0.092), local (*P* = 0.326) or regional (*P* = 0.896) extension of the tumor, or histological grade (*P* = 0.781). Information regarding HPV status was available for 47 of the patients with oropharyngeal carcinomas. The percentage of patients with HPV-positive oropharyngeal carcinomas was 25.5%. No significant differences appeared in PIM-1 expression values as a function of HPV status (*P* = 0.137). Table 1 of the supplementary material shows the median value of the transcriptional expression of PIM-1 according to the variables analyzed.

During the follow-up period, 48 patients (35.6%) had a local recurrence of the tumor, 16 patients (11.9%) had a regional recurrence, and 15 patients (11.1%) had distant metastases. Patients with a local tumor recurrence had significantly higher PIM-1 transcriptional expression values than patients in which treatment with radiotherapy achieved local control of the disease (*P* = 0.017). Figure 1 in the supplementary material shows the distribution of the PIM-1 transcriptional expression values according to the local control of the tumor after treatment with radiotherapy.

**Fig. 1:**
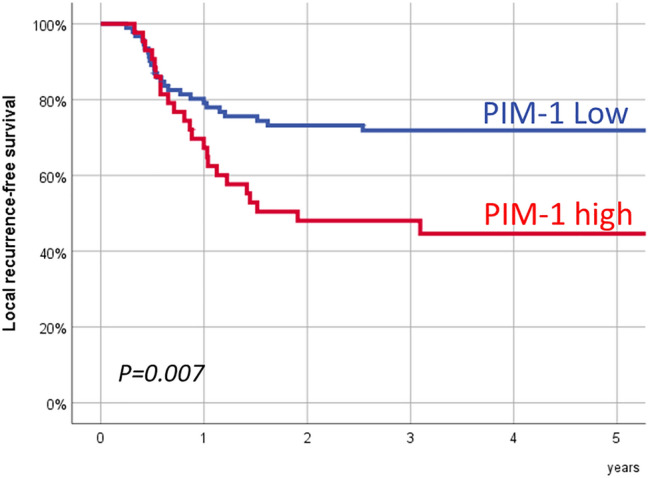
Local recurrence-free survival according to the PIM-1 expression category

The recursive partitioning analysis classified the patients into two groups according to the local control of the tumor after treatment with radiotherapy. Patients with a high PIM-1 expression value (*n* = 43, 31.9%) had a significantly higher rate of local tumor recurrence than patients with low expression (*n* = 92, 68.1%). Five-year local recurrence-free survival for patients with a high PIM-1 expression was 44.6% (95% CI 29.2–60.0%), and for patients with low expression it was 71.9% (95% CI 62.5–81.3%). Figure 1 shows local recurrence-free survival according to the PIM-1 expression categories. There were significant differences in the local recurrence-free survival after treatment with radiotherapy as a function of the PIM-1 expression category (*P* = 0.007).

There were no significant differences in specific-disease survival according to the PIM-1 expression category. Five-year specific-disease survival for patients with a high PIM-1 expression category was 61.1% (95% CI 44.3–77.9%), and for patients with a low expression it was 71.5% (95 CI 62.1–80.9%) (*P* = 0.810). There were also no significant differences in regional (*P* = 0.50) or distant disease (*P* = 0.275) recurrence-free survival as a function of the PIM-1 expression.

Table 2 shows the results of multivariate analysis in which local recurrence-free survival after radiotherapy was considered as the dependent variable. The variables that were associated with an increased risk of local recurrence were the location of the tumor in the hypopharynx, an advanced local extension (cT3–T4), and an elevated PMI-1 transcriptional expression category. Considering those patients with a low expression of PIM-1 as the reference category, patients with high expression had a 2.2-fold increased risk of local recurrence (95% CI 1.22–4.10, *P* = 0.009).

**Table 2:** Results of a multivariate analysis considering the local control after treatment with radiotherapy as the dependent variable

	HR	CI 95% HR	*P*
Location			
Oropharynx	1		
Hypopharynx	2.98	1.14–7.80	0.026
Larynx	1.58	0.64–3.91	0.315
Local extension			
cT1-2	1		
cT3-4	2.92	1.39–6.10	0.004
Regional extension			
cN0	1		
cN +	1.98	0.85–4.61	0.110
Treatment			
Radiotherapy	1		
Chemoradiotherapy	0.95	0.33–2.74	0.930
Bioradiotherapy	1.44	0.52–4.00	0.479
PIM-1			
Low	1		
High	2.24	1.22–4.10	0.009

## Discussion

According to our results, the transcriptional expression of PIM-1 was significantly related to the local control of the tumor in patients with HNSCC treated with radiotherapy, chemoradiotherapy, or bioradiotherapy. Those patients with elevated expression levels of PIM-1 had a 2.24-fold increased risk of local recurrence.

Immunohistochemical studies carried out in patients with HNSCC have found a significant increase in the expression of PIM-1 in tumor tissue, opposite to healthy mucosa [13, 15, 16]. In agreement with the results obtained by other authors that have analyzed the immunohistochemical expression of PIM-1 in HNSCC tumors [13, 15, 16], we did not find a relationship between the transcriptional expression of PIM-1 and clinicopathological variables such as tumor extension or histological grade.

Regarding the prognostic capacity of PIM-1 in patients with HNSCC, the studies performed so far have mostly included patients treated surgically. In a study of 36 patients with oral cavity carcinomas treated with surgery, Chiang et al. [15] found that patients with elevated PIM-1 expression at transcriptional or immunohistochemical level had worse survival, but the differences did not reach statistical significance. Similarly, in a study by Peltola et al. [13] involving 71 patients with HNSCC treated with surgery and/or radiotherapy, those patients with elevated immunohistochemical expression of PIM-1 tended to have poorer survival (*P* = 0.09). Finally, in 39 patients with carcinoma of the tongue treated with surgery, Tanaka et al. [17] found that immunopositivity to PIM-1 correlated with regional and distant recurrences along with a decrease in survival.

In vitro studies performed with HNSCC cell lines have associated PIM-1 with increased tumor aggressiveness. PIM-1 expression has been associated with increased cell proliferation [18, 19], increased migration and invasion capacity [17, 20], and increased cell viability and resistance to apoptosis [18, 19]. In an in vivo experimental study with a murine model, it was shown that inhibition of PIM-1 led to a significant reduction in tumor growth [18]. In nasopharyngeal carcinoma cell lines, PIM-1 inhibition decreased cell proliferation and migration capacity [21].

In tumor models of lung carcinoma [11] or pancreatic carcinoma [12], PIM-1 expression has been associated with a decrease in radiosensitivity. Peltola et al. [13] observed that in patients with HNSCC treated with preoperative radiotherapy, a high expression of PIM-1 correlated with a reduced response to radiotherapy. In HNSCC cell lines, these authors demonstrated that inhibition of PIM-1 expression with siRNA induced radiosensitization of tumor cells, therefore supporting the concept that PIM-1 protects cells from radiotherapy-induced damage. Similarly, in prostate carcinoma cell lines it has been shown that the increased sensitivity to radiotherapy mediated by miR-124 and miR-144 was a consequence of PIM-1 downregulation [22].

In addition, an association has been described between PMI-1 expression and resistance to chemotherapy in prostate carcinoma models [23]. There is also evidence that downregulation of PIM-1 activity increased sensitivity to chemotherapy in the colon [24] or bladder carcinoma cell lines [25].

In our study, we found a significant relationship between PIM-1 transcriptional expression and the risk of local recurrence in patients treated with radiotherapy, including patients treated with chemoradiotherapy and bioradiotherapy. Figure 2 of the supplementary material shows the local recurrence-free survival as a function of the PIM-1 expression category depending on the type of treatment used. According to the results of multivariate analysis, the variables that were significantly associated with an increased risk of local recurrence after radiotherapy were hypopharyngeal tumor location, advanced local extension (cT3-4), and high PIM-1 expression.

These differences in local disease control were not ultimately reflected in specific survival, basically due to the possibility of carrying out salvage surgeries after a local recurrence of the tumor. Salvage surgery entails a higher frequency of postoperative complications and morbidity versus similar surgery performed as an initial treatment. The availability of a biomarker with the capacity to assess the sensitivity to radiotherapy would make it possible to personalize the type of treatment according to the tumor's sensitivity profile.

One of the limitations of our study was that it only assessed the transcriptional expression of PIM-1, without providing information about the signaling pathways activated or the existence of post-transcriptional regulation mechanisms. In addition, these results were obtained retrospectively in a single institution from a heterogeneous sample of patients in terms of the location of the primary tumor and the type of treatment used, including patients treated with chemo- and bioradiotherapy. Independent validation of our results would be required before considering PIM-1 transcriptional expression as a predictive biomarker of response to radiotherapy in patients with HNSCC.

## Conclusion

PIM-1 transcriptional expression was significantly related to local disease control in patients with HNSCC treated with radiotherapy. Patients with elevated expression levels of PIM-1 had a significantly higher risk of local recurrence.

## Supplementary Information

Below is the link to the electronic supplementary material.

Supplementary file1 (DOCX 151 KB)
