# Supplementary material for: Relationship between the transcriptional expression of PIM1 and local control in patients with head and neck squamous cell carcinomas treated with radiotherapy
Source: Eur Arch Otorhinolaryngol. 2022 Jan 6;279(7):3679–84. doi: 10.1007/s00405-021-07223-4 (PMC9130163; doi:10.1007/s00405-021-07223-4)

**Supplementary material**

**Supplementary table 1**. Median of the transcriptional expression values of the PIMP-1 according to several clinical variables.

|  |  | **Median PMI-1 expression** | **P** |
| --- | --- | --- | --- |
| **Sex** | Male | 46.5 | 0.941 |
|  | Female | 44.2 |  |
| **Age** | <65 years | 48.1 | 0.060 |
|  | ≥65 years | 40.1 |  |
| **Toxics** | None | 49.2 | 0.301 |
|  | Moderate | 34.4 |  |
|  | Severe | 46.7 |  |
| **Location** | Oropharynx | 48.3 | 0.092 |
|  | Hypopharynx | 31.9 |  |
|  | Larynx | 47.8 |  |
| **T classification** | cT1-2 | 43.0 | 0.326 |
|  | cT3-4 | 47.6 |  |
| **N classification** | cN0 | 47.6 | 0.896 |
|  | cN+ | 45.3 |  |
| **Histologic grade** | Well | 49.2 | 0.781 |
|  | Moderate | 46.5 |  |
|  | Poor | 41.8 |  |
| **HPV*** | Negative | 51.2 | 0.137 |
|  | Positive | 325 |  |

* 47 patients with oropharyngeal carcinoma.

**Supplementary figure 1.** Distribution of the transcriptional expression values of PIM-1 according to the local control of the tumor after treatment with radiotherapy.


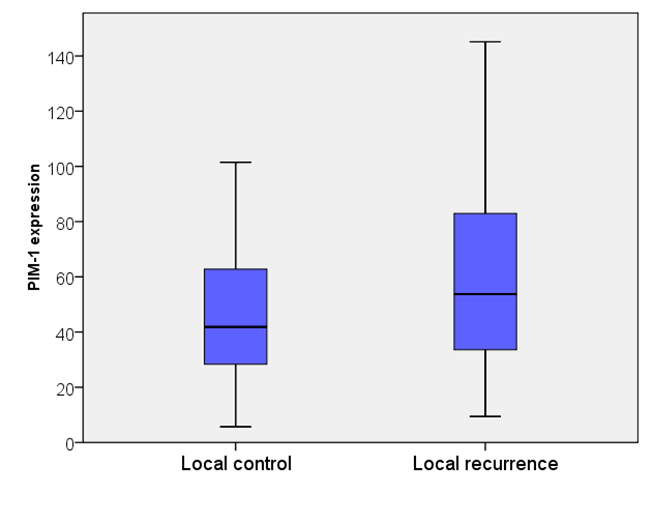


**Supplementary figure 2.** Local recurrence-free survival as a function of the PIM-1 expression category depending on the type of treatment.


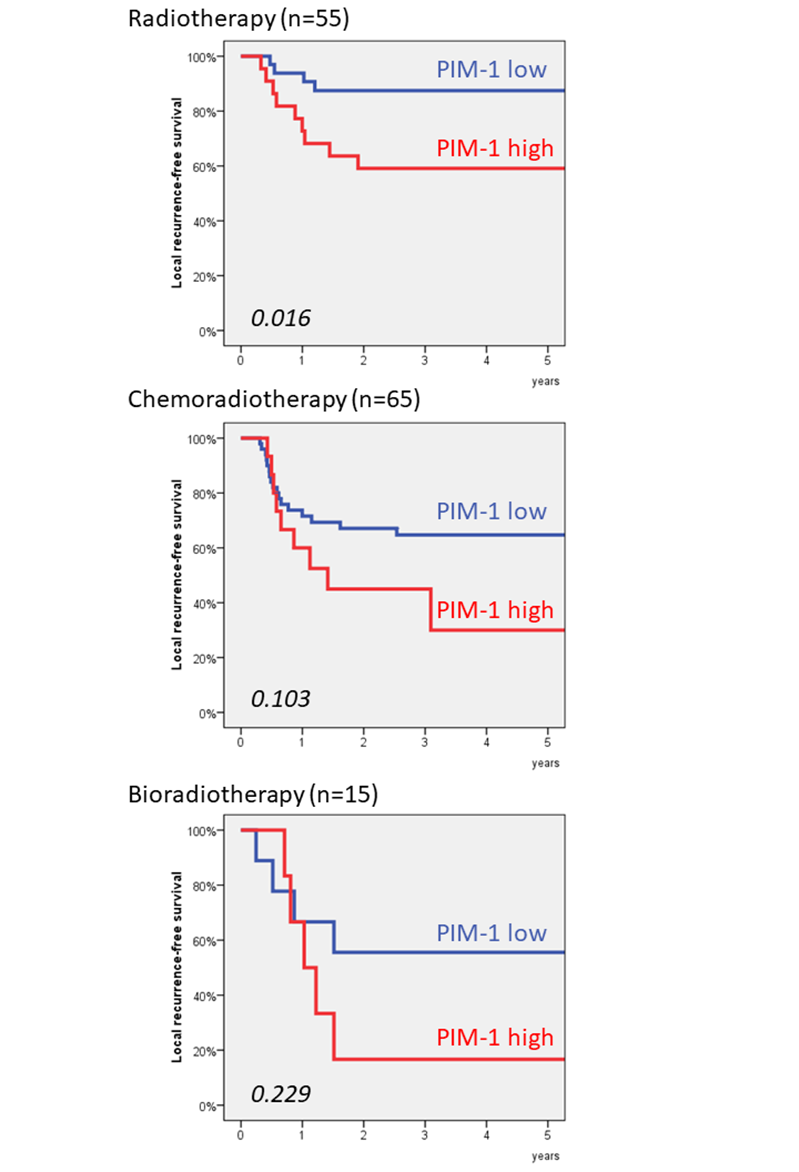

Supplement: Supplementary file 1 — Supplementary file1 (DOCX 151 KB) [file 405_2021_7223_MOESM1_ESM.docx]
